# Quantitative MRI susceptibility mapping reveals cortical signatures of changes in iron, calcium and zinc in malformations of cortical development in children with drug-resistant epilepsy

**DOI:** 10.1016/j.neuroimage.2021.118102

**Published:** 2021-09

**Authors:** Sara Lorio, Jan Sedlacik, Po-Wah So, Harold G. Parkes, Roxana Gunny, Ulrike Löbel, Yao-Feng Li, Olumide Ogunbiyi, Talisa Mistry, Emma Dixon, Sophie Adler, J. Helen Cross, Torsten Baldeweg, Thomas S. Jacques, Karin Shmueli, David W Carmichael

**Affiliations:** a Developmental Neurosciences, Great Ormond Street Institute of Child Health, University College London, London, UK; b Wellcome EPSRC Centre for Medical Engineering, School of Biomedical Engineering and Imaging Sciences, King's College London, St Thomas’ Hospital, London SE1 7EH, UK; c Biomedical Engineering Department, School of Biomedical Engineering & Imaging Sciences, King's College London, London, UK; d Center for the Developing Brain, School of Biomedical Engineering and Imaging Sciences, King's College London, London, UK; e Department of Neuroimaging, Maurice Wohl Clinical Neuroscience Institute, Institute of Psychiatry, Psychology and Neuroscience, King's College London, UK; f Department of Radiology, Great Ormond Street Hospital for Children NHS Foundation Trust, London, UK; g Developmental Biology and Cancer Programme, UCL Great Ormond Street Institute of Child Health, University College London and Department of Histopathology, Great Ormond Street Hospital for Children NHS Foundation Trust, London, UK; h Pathology Department, Tri-Service General Hospital and National Defence Medical Centre, Taipei, Taiwan, ROC; i MRI Group, Department of Medical Physics and Biomedical Engineering, University College London, London, UK

**Keywords:** Focal cortical dysplasia, Malformation of cortical development, Quantitative magnetic susceptibility, Drug-resistant epilepsy, Brain mineral content

## Abstract

**Objective:**

Malformations of cortical development (MCD), including focal cortical dysplasia (FCD), are the most common cause of drug-resistant focal epilepsy in children. Histopathological lesion characterisation demonstrates abnormal cell types and lamination, alterations in myelin (typically co-localised with iron), and sometimes calcification. Quantitative susceptibility mapping (QSM) is an emerging MRI technique that measures tissue magnetic susceptibility (χ) reflecting it's mineral composition.

We used QSM to investigate abnormal tissue composition in a group of children with focal epilepsy with comparison to effective transverse relaxation rate (R2*) and Synchrotron radiation X-ray fluorescence (SRXRF) elemental maps. Our primary hypothesis was that reductions in χ would be found in FCD lesions, resulting from alterations in their iron and calcium content. We also evaluated deep grey matter nuclei for changes in χ with age.

**Methods:**

QSM and R2* maps were calculated for 40 paediatric patients with suspected MCD (18 histologically confirmed) and 17 age-matched controls.

Patients’ sub-groups were defined based on concordant electro-clinical or histopathology data. Quantitative investigation of QSM and R2* was performed within lesions, using a surface-based approach with comparison to homologous regions, and within deep brain regions using a voxel-based approach with regional values modelled with age and epilepsy as covariates.

Synchrotron radiation X-ray fluorescence (SRXRF) was performed on brain tissue resected from 4 patients to map changes in iron, calcium and zinc and relate them to MRI parameters.

**Results:**

Compared to fluid‐attenuated inversion recovery (FLAIR) or T1‐weighted imaging, QSM improved lesion conspicuity in 5% of patients.

In patients with well-localised lesions, quantitative profiling demonstrated decreased χ, but not R2*, across cortical depth with respect to the homologous regions. Contra-lateral homologous regions additionally exhibited increased χ at 2–3 mm cortical depth that was absent in lesions. The iron decrease measured by the SRXRF in FCDIIb lesions was in agreement with myelin reduction observed by Luxol Fast Blue histochemical staining.

SRXRF analysis in two FCDIIb tissue samples showed increased zinc and calcium in one patient, and decreased iron in the brain region exhibiting low χ and high R2* in both patients. QSM revealed expected age-related changes in the striatum nuclei, substantia nigra, sub-thalamic and red nucleus.

**Conclusion:**

QSM non-invasively revealed cortical/sub-cortical tissue alterations in MCD lesions and in particular that χ changes in FCDIIb lesions were consistent with reduced iron, co-localised with low myelin and increased calcium and zinc content. These findings suggest that measurements of cortical χ could be used to characterise tissue properties non-invasively in epilepsy lesions.

## 1 Introduction

Malformations of cortical development (MCD), and in particular focal cortical dysplasia (FCD), are the most common cause of drug-resistant focal epilepsy in children (Blumcke et al., 2017; Harvey et al., 2008). FCD is characterised by a broad spectrum of histopathological abnormalities that are used to classify lesions in different sub-types. According to the classification system developed by the International League Against Epilepsy, the presence of disrupted radial or tangential cortical lamination is associated with FCDI lesions, dysmorphic neurons characterise FCDIIa, and dysmorphic neurons combined with balloon cells in the cortex and white matter occur in FCDIIb (Blümcke et al., 2011). These neuro-pathological features could be the result of a developmental abnormality involving several phases of corticogenesis (Zucca et al., 2015).

Iron is involved in many fundamental neural processes such as myelin production, oxygen transportation as well as synthesis and metabolism of neurotransmitters (Ward et al., 2014). Moreover iron accumulation in deep brain regions characterises brain ageing (Acosta-Cabronero et al., 2016; Aquino et al., 2009; Ashraf et al., 2018; Ghassaban et al., 2019; Hallgren and Sourander, 1958; Li et al., 2014; Yao et al., 2009) and drug-resistant epilepsy could potentially hasten this process.

Previous studies have shown that FCDIIb lesions exhibit decreased myelin ([Bibr bib0012][Bibr bib0011]). Myelin plays an important role in the development and function of neocortex, which is characterised by a layered structure with region-specific myeloarchitecture and cytoarchitecture (Dinse et al., 2015; Fatterpekar et al., 2002; Timmler and Simons, 2019; Waehnert et al., 2014). Although myeloarchitecture varies across cortical regions, in deep cortical layers there is a consistent pattern of high myelination co-localising with increased laminar iron content that has been observed with MRI (Deistung et al., 2013b; Fukunaga et al., 2010). This pattern of co-localisation is expected to be disrupted in the presence of altered laminar structure and malformations of cortical development, based on the typical radiological signature of blurring at the border between WM and GM together with pathological changes in myelination (Blumcke et al., 2017).

Calcification and iron accumulation occurs in numerous neurological disorders with possible neurotoxic consequences such as oxidative damage and cell death (Casanova and Araque, 2003). Although it remains unclear the extent to which FCD lesions have altered levels of calcium and iron (Aggarwal et al., 2018), tissue property changes underlying epilepsy have been widely investigated using animal models that have shown calcification and oxidative stress markers in epileptogenic regions (Gayoso et al., 2003; Löscher, 2011; Zimmer et al., 2019). Whilst the mechanisms underlying mineral and metal accumulation are not yet fully understood, measuring altered chemical composition in FCD lesions non-invasively may provide an important biomarker for local pathological processes.

Currently, the non-invasive techniques employed to assess the presence of metal and mineral content in the brain are measurements of the effective transverse relaxation rate (R2*=1/T2*) and more recently, quantitative susceptibility mapping (QSM). R2* is sensitive to both macroscopic and microscopic magnetic field inhomogeneities, the latter may occur due to the presence of iron or myelin in tissue (Homayoon et al., 2019; Ning et al., 2014; Ordidge et al., 1994; Tabelow et al., 2019; Yao et al., 2009). QSM provides a quantitative and local anatomical contrast by estimating the magnetic susceptibility (χ) using the MRI signal phase (de Rochefort et al., 2010; Deistung et al., 2017; Haacke et al., 2015, p. 201; Liu et al., 2015a; Shmueli et al., 2009). Typically, in the human brain a strong χ image contrast is obtained in structures containing paramagnetic substances, such as iron-rich deep brain nuclei showing positive χ values tissue (Bilgic et al., 2012; Zheng et al., 2013), and in regions with diamagnetic substances, such as highly myelinated white matter fibres and calcifications exhibiting negative χ values (Langkammer et al., 2012; Li et al., 2011). Therefore, we would expect QSM to be sensitive to the calcium alterations in lesions and to the disruption of cortical myeloarchitecture.

Both χ and R2* values are sensitive to age-related changes of iron in deep grey matter nuclei (Yao et al., 2009), but χ can, unlike R2*, distinguish between sources of paramagnetic and diamagnetic substances. For this reason QSM has been used clinically to differentiate between haemorrhages and calcifications (Klohs et al., 2011; Liu et al., 2012; Lotfipour et al., 2012; Wisnieff et al., 2015; Deistung et al., 2013b; Chen et al., 2014). However, the only application of QSM to focal epilepsy so far has been limited to animal models (Aggarwal et al., 2018).

The aim of this study is the non-invasive characterisation of abnormal tissue mineral composition using QSM and R2* in a group of children with focal epilepsy. Therefore QSM mapping was performed in patients with MRI visible lesions consistent with malformations of cortical development. Our primary hypothesis was that *local* reductions in χ could be found in lesions due to underlying alterations in iron or calcium content. This hypothesis was investigated in a group of subjects by evaluating χ changes radiologically and quantitatively. We investigated changes in the expected laminar distribution of cortical iron at the group level using a surface-based approach, sampling χ at increasing depths from the pial surface within lesions and homologous regions. In a small group, χ changes were compared to synchrotron radiation X-ray fluorescence (SRXRF) elemental measurements of iron, calcium and zinc content (Carver et al., 2016; Coulter, 2000; Zheng et al., 2013).

Our secondary hypothesis was that there could be regional brain iron changes in the deep grey matter nuclei in children with poorly controlled epilepsy, reflecting the patho-physiological changes within extended epileptic networks (Centeno et al., 2014; He et al., 2017). To test this, we measured age-changes in χ within deep brain nuclei of healthy controls and assessed if this relationship was different in subjects with focal epilepsy. The same analysis was repeated with the R2* measurements.

## 2 Materials and methods

### 2.1 Participants

A retrospective cohort of 40 patients (mean age =8.8 ± 5 years, range=2–21 years, female=16) was identified for this research study from all those undergoing assessment for epilepsy surgery at Great Ormond Street Hospital (GOSH), following approval by the national research ethics service. Patients were included if they had a diagnosis of suspected MCD based on radiological and electro-clinical reports. This meant that all patients had identifiable lesions on MRI. All patients underwent video-telemetry–EEG to document seizures and 3T MRI at GOSH with the standard epilepsy imaging protocol. Patients were excluded if MRI scans showed severe motion artefacts (i.e. indistinguishable adjacent gyri due to motion or severe ringing), they were younger than 2 years of age, or they were without the full MRI protocol described in the following section.

From the full cohort of 40 patients, sub-groups with *n* ≥ 10 patients were identified to perform group analysis.

Additionally, 17 healthy controls (mean age = 15 ± 3 years, range= 8–21 years, female=10) were scanned for this research study at Great Ormond Street Hospital.

### 2.2 MRI protocol

All patients were scanned on a 3T whole-body MRI system (Magnetom Prisma, Siemens Medical Systems, Germany), using a 20-channel receive head coil and body coil for transmission. The clinical protocol included 3D T1-weighted (T1w) images acquired using magnetisation prepared rapid gradient echo (MPRAGE) (repetition time (TR)=2300 ms, echo time (TE)=2.74 ms, matrix size 256 × 208 × 256, flip angle=8°, voxel size=1 × 1 × 1mm^3^), 3D FLAIR (TR/TE/inversion time/ flip angle: 4000 ms/395 ms/1800 ms/120°, matrix size=306 × 192 × 384, voxel size=0.65 × 1 × 0.65mm^3^), and three-dimensional T2*-weighted gradient echo images used to compute R2* and QSM (TR=38 ms, flip angle=15 deg). The T2*-weighted images were acquired with a monopolar multi-echo acquisition at seven equidistant TE between 3 and 27 ms, resolution=1.15 × 1.15 × 1.15mm^3^, matrix size 156 × 192 × 144, and approximately 6 min acquisition time. Parallel imaging was used along the phase-encoding (PE) direction (acceleration factor 2 GRAPPA reconstruction), 6/8 partial Fourier was applied in the partition direction. This sequence was also used for computing susceptibility-weighted images (SWI) with minimum intensity projection (mIP). The SWI and mIP were included in the clinical protocol in order to provide state-of-the-art evaluation of vessel micro-bleeds and vascular abnormalities that are common in this cohort(Craven et al., 2012).

Healthy controls were scanned on a 3T whole-body MRI system (Magnetom Prisma, Siemens Medical Systems, Germany), using a 64-channel receive head coil and body coil for transmission. The scanning protocol and acquisition parameters were the same as the ones used for the patients’ data acquisition.

### 2.3 Calculation of QSM and R2* maps

To reconstruct the QSM map, an initial brain mask was calculated based on the first TE magnitude image, to avoid mask errors in regions with short T2*, using the Brain Extraction Tool (Smith, 2002) from FSL6.0. The initial brain mask was iteratively eroded in border areas with too high (>97.5%) or too low (<2.5%) phase values in the local phase map, as these phase values were considered artefacts. The iteration stopped when the standard deviation of the phase values of the border of the mask was below 1.2 times of the standard deviation of the phase values within the mask. The iteration also stopped, if the local mask area was smaller than 70% of the initial mask.

The processing pipeline for QSM consisted of: non-linear fitting of the complex GRE signal over multiple echoes (Liu et al., 2015a); spatial phase unwrapping using Laplacian kernels (Schweser et al., 2013) and background field removal using the Laplacian boundary value method (Zhou et al., 2014). The local field-to-susceptibility inversion was performed by applying direct Tikhonov regularization (Kressler et al., 2010), and correction for the susceptibility underestimation (Schweser et al., 2013). The correction for susceptibility underestimation involved multiplying every susceptibility map by a constant correction factor. This factor was calculated from the point-spread function of the modified dipole kernel used for QSM calculation (Schweser et al., 2013). For the Tikhonov-based regularization, the optimal regularization factor (α = 0.06) was calculated as the average across 10 representative subjects of the individual optimal regularization parameters calculated using the L-curve method (Chary et al., 2021, Hansen and O'Leary, 1993). Susceptibility mapping data processing is an area of active research and new methods might improve the sensitivity and specificity of the results presented here.

R2* maps were computed using a voxel-wise linear fit of the logarithm of the magnitude image over the TE of the T2*-weighted dataset.

### 2.4 Visual assessment

To evaluate the lesion visibility on the QSM and R2* relative to the clinical images, two neuro-radiologists (RG, UL) were presented with coregistered T1w, FLAIR, QSM and R2* images. Following standard radiological practice, they were allowed to compare between the different contrasts in the same patient; this allowed them to visually assess lesion location and relative conspicuity in the different image types. A lesion visibility score from 1 to 4 (1=not visible, 2=subtle, 3=visible, 4=clearly visible) was assigned to each image type for each patient. The observers were not blinded to the radiological and EEG reports (i.e.: localisation of the lesion) because the aim was to derive relative conspicuity not test lesion detection performance. Intensity windowing was individually adapted to gain optimal contrast. The neuro-radiologists assessed the images independently and so were blinded to each other's ratings.

To compare lesion conspicuity scores between T1w, FLAIR, R2* and QSM images across patients, we applied Friedman test to the mean score of the two radiologists computed for each map. Then we performed a multiple comparison test between the ranking means provided by the Friedman test for each group of images. We set statistical significance at *p*<0.05 after applying the Bonferroni correction for multiple comparisons.

### 2.5 Image processing

#### 2.5.1 Sampling of cortex and subcortical white matter

**Fig. 1:**
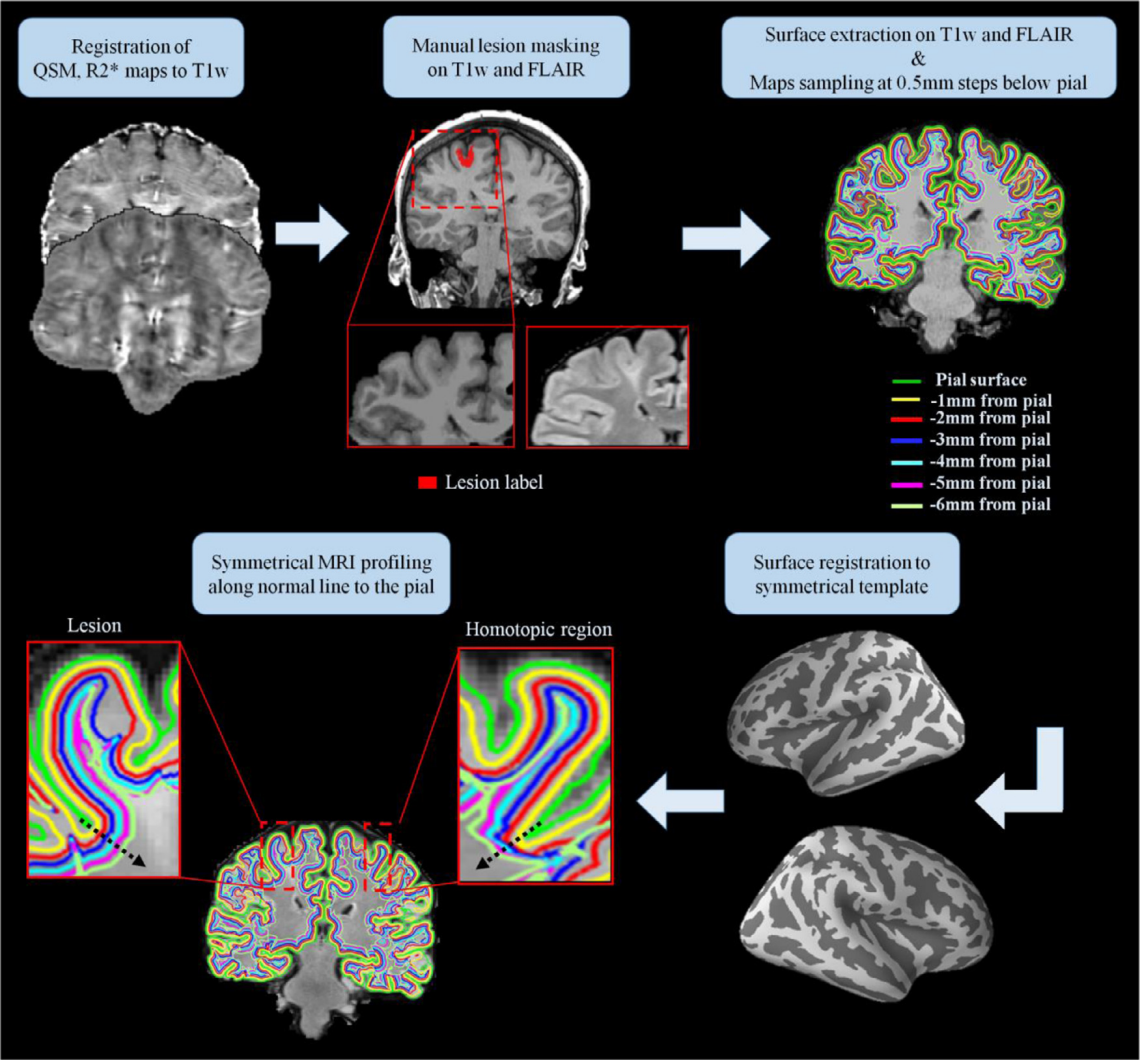
Maps sampling workflow. First the QSM and R2* maps were coregistered to the T1-weighted (T1w) data. Manual lesion masking and surface extraction at increasing depths were performed on T1w and FLAIR images. The QSM and R2* maps were projected onto the surfaces and sampled at several depths from the pial surface. The sampled surfaces and the lesion masks were then spatially registered to a symmetric template, allowing the symmetrical MRI profiling of the QSM and R2* maps along the normal (black arrow) to the pial surface for each lesion and the corresponding homotopic region.

Fig. 1 shows the workflow for the sampling of the QSM and R2* data. FreeSurfer software v5.3 (Dale et al., 1999; Fischl and Dale, 2000) was used to co-register the QSM, R2* maps and FLAIR to the T1w image, and to reconstruct cortical and subcortical surfaces. In order to avoid co-registration errors due to the contrast difference between QSM and T1w, first we co-registered the SWI mean magnitude, computed across the echo points, to the T1w image. Then the spatial transformation matrix was applied to the QSM and R2*.

Both FLAIR and T1w images were employed to generate the surface reconstruction. The image processing steps included skull-stripping (Ségonne et al., 2004), intensity non-uniformity correction, automated Talairach transformation, extraction of the deep GM structures (including hippocampus, amygdala, caudate, putamen, ventricles) (Fischl et al., 2004), and intensity normalisation (Sled et al., 1998). Finally the images were tessellated and deformed to create accurate smooth mesh representations of the pial surface (Fischl and Dale, 2000; Ségonne et al., 2007).

To examine the cortical and subcortical signal of the QSM and R2* maps while minimising GM-WM misclassification errors, the QSM and R2* maps were sampled along surfaces extracted at different depths from the pial surface down to 6 mm at steps of 0.5 mm. This avoided the potential confounds related to identifying the white/grey matter border in FCD where often no clear border exists, and which can cause the cortical thickness to be mis-estimated.

The QSM and R2* surfaces were smoothed using a 10 mm vertex-wise FWHM Gaussian kernel in order to increase stability of the per‐vertex sampling at different depths (Adler et al., 2017b; Jin et al., 2018; Lorio et al., 2020; Wagstyl et al., 2020). Manual delineated lesions had a median area of 1074 mm^2^ and median absolute deviation of 756 mm^2^, which is much larger than these smoothing kernels.

Then smoothed QSM and R2* surfaces were registered to an average symmetric space having an identical number of vertices for each hemisphere (Greve et al., 2013). This allowed us to analyse the χ and R2* profile changes between homologous regions, guided by a straight line orthogonal to the cortical surface that provided vertex correspondence across surfaces (Polimeni et al., 2010). While this does not fully account for variable cortical thickness, it allows using homologous vertexes as internal controls where the contra-lateral region would be expected to have comparable thickness in the absence of pathology.

#### 2.5.2 Lesion masking

FCD lesions were delineated on T1w and FLAIR images only by the most experienced paediatric neuro-radiologist (R.G.). 3D binary masks were manually delineated for the 40 patients. The lesion masks were first registered onto the FreeSurfer surface reconstructions and then to the symmetric template as shown on Fig. 1. This procedure provided a mask for the lesion and one for the homologous healthy tissue.

#### 2.5.3 Segmentation of deep brain regions

To perform age regression models using QSM and R2* maps, deep brain nuclei characterised by high iron content, were delineated using atlas based masks (Lorio et al., 2016). The regions analysed were the globus pallidus, putamen, caudate, thalamus, substantia nigra, subthalamic nucleus, red nucleus and cerebellar dentate. The masks were warped from the MNI space to the subject's native space using the deformation fields estimated with SPM12 and Matlab 9.5 (Mathworks, Sherborn, MA, USA). The deformation fields were computed by performing tissue classification on the T1w images using the “segmentation” approach in SPM12 (Ashburner and Friston, 2005). The suspected FCD patients did not exhibit any lesions in the deep brain nuclei.

### 2.6 Statistical analysis

#### 2.6.1 Lesion profiling

MRI profiles for the QSM and R2* maps were obtained by averaging the values within each patient's masks for the lesion and homologous region, separately, along cortical and subcortical surfaces. To investigate signal changes in FCD lesions, QSM and R2* profiles were statistically compared between lesion and homologous regions for the 40 patients. The same analysis was repeated within the sub-group of patients with FCD IIb, and within the well-localised sub-group.

We used a paired *t*-test to evaluate χ and R2* differences at various sampling depths. Correction for multiple comparisons was applied using false discovery rate (FDR) at *q*<0.05. Data normality, required for the *t*-test, was assessed using the Shapiro-Wilk test run on the MRI profiles of the lesions and homologous regions.

#### 2.6.2 Age effect in deep brain nuclei

To test for a linear correlation of χ and R2* values with age, a multiple regression model was applied to both the whole cohort of patients and controls. The same analysis was repeated on the controls and the following sub-groups: 15 patients with FCDIIb and 25 patients with well-localised lesions.

For each deep brain structure the mean χ and R2* was modelled using age, disease duration and disease presence as independent variables. The regression was implemented using the ordinary least square method embedded in IBM SPSS v26. In order to test for the interaction between age and disease presence on the χ and R2* respectively, a hierarchical multiple regression was applied. This model assessed the increase in variance explained by the addition of the interaction term between age and disease presence to the main effects model.

Additionally, partial correlation coefficients of the age and disease duration with the mean χ, and R2* respectively, were estimated for the whole cohort of patients. The partial correlation coefficients were computed by removing the correlation that was due to the association between age and disease duration using IBM SPSS v26.

### 2.7 SRXRF elemental mapping and analysis

To identify the elements driving QSM changes in the FCD lesions, we used SRXRF to simultaneously quantify and spatially evaluate elements in the brain tissue resected during surgery. One subject without diagnostic findings and three with different pathologies were selected on the basis of having clear visually apparent changes in QSM maps within the lesion and recovery of sufficient amounts of tissue from surgical resection. In order to allow the registration of the specimen with the MRI maps, we selected tissue samples containing white and grey matter with partially preserved anatomical borders.

Each brain specimen was placed in formalin shortly after surgery and processed for embedding in wax for histological examination using standard diagnostic testing that included haematoxylin and eosin (H&E) staining, Luxol Fast Blue/Cresyl Violet staining (LFB) and immunohistochemistry as required for diagnosis. The detailed description of staining techniques applied is reported in the Supplementary material.

Microtomy was performed to produce 7 μm thick formalin fixed paraffin embedded sections that were mounted onto 4 μm thick Ultralene film (Spex Sample-Prep, NJ, USA) secured to a customized holder for SRXRF.

SRXRF of the brain tissue sections was performed on the I18 beam-line at the Diamond Light Source synchrotron radiation facility (Didcot, UK) as described by Walker and colleagues (Walker et al., 2016). Briefly, the beam energy was tuned to 11 keV and focused to 100 × 100 μm (determining resolution) and the tissue samples scanned in a raster manner. The samples were mounted at a 45° angle with respect to the incoming X-ray beam and the detector to minimize scatter contribution. The raw data consist of full energy dispersive spectra for each sample point exposed to the beam. The spectra were subsequently fitted using PyMca (Solé et al., 2007) and the areas of the characteristic peaks for iron, zinc and calcium evaluated. Quantification was performed by measuring a reference metal film (AXO, GmbH, Dresden, Germany) to estimate photon flux on the samples (Walker et al., 2016). Regions of interest were manually drawn on the SRXRF maps of pixel-by-pixel elemental iron, zinc and calcium concentrations (parts per million, ppm), and were used to quantify iron, zinc and calcium (mean±standard deviation) using ImageJ (https://imagej.nih.gov/ij/).

In order to enable qualitative comparison between SRXRF elemental maps, histology and MRI data, the digitized histological sections were visually inspected by an experienced observer (YFL) and anatomical landmarks were identified to allow the manual alignment of histology staining and elemental maps using ImageJ. The FLAIR images from all cases were visually compared with the elemental maps, and the best-fitting MRI slices were chosen using anatomical landmarks, such as gyri, sulci, and grey, white matter tissue portions to manually align the elemental maps. Neighbouring normal tissue for comparison was chosen within the tissue block available based on the following criterion; the absence of pathological features including dysmorphic neurons, balloon cells, abnormal mineral content and gliosis, based on visual assessment by expert observers (TJ, YFL).

### 2.8 Data availability

Patients’ main demographic and clinical data are available in Table 1. Other data, including brain imaging data, are available upon reasonable request, but cannot be made open because of ethics protocol requirement and the sensitive nature of patient data.

**Table 1:** Patient demographics.

Patient	Gender (*m* = 0)	Age (y)	Disease onset	MRI		EEG		Histology	Surgery	Engel
				site	radiological report	localising	site			
										
1	0	3	22m	R mesial par-occ	focal signal abnormality	y	R post	Glioneuronal tumour	y	Ia
2	0	16	3y	R inf fr gyrus, fr operculum	focal signal abnormality	y	R fr	FCD IIb	y	Ia
3	0	2	1y	L fr, sup longitudinal sulcus	subtle lesion	n		NA	n	
4	1	8	2y7m	L par operculum	abnormal cortical folding, blurring	y	L side predominance	FCD IIb	y	III/IV
5	0	4	3y	R post fr, ant motor strip	blurring	y	R post temp lobe	FCD IIa	y	Ia
6	0	15	2m	L paracentral lobule	focal signal abnormality	y	L paracentral lobule	FCD IIb	y	Ia
7	1	16	6y	L sup fr gyrus	focal signal abnormality	y	L front area	No abnormality	y	Ia
				L mesial temp	hippocampal sclerosis			HS (ILAE 3)		
8	0	9	11m	L sup fr gyrus	focal signal abnormality	y	L frontotemporal	FCD IIb	y	II
9	0	9	3d	R fr, ant temp	smaller hemisphere, diffuse blurring	y	R fr	FCD IIb	y	II
10	0	8	8y	R post temp	focal signal abnormality, transmantle sign	n		NA	n	
11	0	4	3y	R temp	focal signal abnormality	n	diffuse	NA	n	
12	0	11	3m	R par-occ	blurring	n	multifocal	NA	n	
13	1	17	1y	L post cingulate gyrus	focal signal abnormality	n		No abnormality	y	Ia
				L mesial temp	hippocampal sclerosis			HS (ILAE 1)		
14	1	7	9m	R middle, inf fr, sup par gyri	focal signal abnormality	n	diffuse	NA	n	
15	0	18	1y	L inf temp sulcus	focal signal abnormality	y	temp and fr both sides	FCD IIb	y	Ia
16	1	11	3w	L temp	white matter signal change	y	L paracentral	NA	n	
17	0	17	9y	L fr gyrus	focal signal abnormality	n		NA	n	
18	0	11	2y	L sup temp gyrus	focal signal abnormality	y	L temp	FCD IIb	y	Ia
19	0	16	12y	L occ	subtle difference in grey-white matter differentiation	n	multifocal fronto-parietal	NA	n	
20	0	14	2m	L paracentral lobule	focal signal abnormality	y	L paracentral	FCD IIb	y	Ia
21	0	2	2m	L occ	focal signal abnormality	y	L posterior quad	NA	n	
22	1	7	6y	L temp	focal signal abnormality	y	L post temp lobe	glioneuronal tumour	y	Ia
23	0	1	9m	L fr	focal signal abnormality	y	L fr	NA	n	
24	1	6	23m	L fr	blurring	y	R fr onset and L fronto-temporal	FCD IIa	y	III/IV
25	1	4	8w	L sup temp gyrus	focal signal abnormality	y	Left temporal	FCD IIb	y	Ia
26	1	2	13m	R middle temp gyrus	focal signal abnormality	y	Right temporal posterior	FCD IIb	y	Ia
27	0	12	1.5y	R temp	hippocampal sclerosis			HS (ILAE 1)	y	III/IV
				R middle temp gyrus	focal signal abnormality	y	R temp lobe	No abnormality		
28	0	13	6y	R fr and insular lobes	focal signal abnormality	y	R fr lobe	Polymicrogyria	y	Ia
29	0	6	7m	R int and mesial fr lobe	blurring	n		NA	n	
30	1	15	11y	L precentral gyrus	focal signal abnormality	n		NA	n	
31	0	10	3m	R par and occ	blurring	n	Multifocal	NA	n	
32	0	10	9w	L post insular region	white matter signal change	y	(Stereoeeg) L insula	NA due to laser ablation	y	Ia
33	0	10	7y	R sup and middle fr gyri	focal signal abnormality	n		NA	n	
34	1	15	14y	L temp	subcortical lesion	y	L ant cortical	NA	n	
35	1	2	8m	R fr	focal signal abnormality	y	R fr	FCD IIa	y	Ia
36	1	6	1y	L fr	focal signal abnormality	y	L fr	FCD IIb	y	II
37	1	5	6.5m	R occ	focal signal abnormality	y	R post-temp	FCD IIb	y	Ia
38	1	6	1y	R fr	focal signal abnormality	y	R ant	FCD IIb	y	Ia
39	0	2	1y	L temp	focal signal abnormality	y	L temp	FCD IIb	y	Ia
40	0	6	2y	R fr	focal signal abnormality	y	R mostly fr	FCD IIb	y	Ia

Age is presented in years, while seizure onset is presented in years (y), months (m) or weeks (w). The lesion location is reported according to the MRI-based radiological report. Electrophysiological clinical information is provided based on the EEG seizure localisation. If multiple lesion locations were reported in patients who underwent surgery, all locations were resected. Surgery outcome for the patients who underwent surgery is classified using the Engel post-operative surgical outcome according to Engel classification (Engel, 1993), Ia = completely seizure free, III = worthwhile improvement, IV = no worthwhile improvement. Abbreviations: *m*=male, *f*=female, *L*=left, *R*=right, fr= frontal pole, ant= anterior, inf= inferior, occ=occipital pole, par=parietal pole, post=posterior, sup=superior, temp=temporal lobe, FCD=focal cortical dysplasia, HS=hippocampal sclerosis, *n*=no, *y*=yes.

## 3 Results

### 3.1 Patients’ clinical information

The patients’ demographics and clinical information can be found in Table 1. A total of 25/40 patients underwent surgery: 18 were histologically diagnosed with FCD (15 FCDIIb, 3 FCDIIa, 16 had Engel outcome ≥II), one had laser ablation, therefore the histological examination was not possible, one had polymicrogyria, two had glioneuronal tumours, three were diagnosed with hippocampal sclerosis (HS) and had standard temporal lobe resection involving the anterior temporal lobe, histopathological analysis did not exhibit any specific diagnostic pathology outside the hippocampus in these cases (Table 1). Seizure freedom was achieved in 19/25 cases at a mean of 1.5 years after surgery.

We identified two sub-groups with *n* ≥ 10 patients: the one with 15 FCDIIb patients and the group of patients with well-localised lesions. The criteria for including patients in the well-localised group were as follows: having resection inclusive of the MRI defined lesion with histopathological confirmation, or presenting with an MRI-visible lesion concordant with the electro-clinical data. The well-localised lesions group was composed of 25 patients: 21 that received a histopathological diagnosis following epilepsy surgery (15 FCDIIb, 3 FCDIIa, 1 polymicrogyria, 2 Glioneuronal tumours), and 4 patients without surgery, having the MRI-visible lesion concordant with the electro-clinical data. Patients diagnosed with HS were not included in the well-localised group due to the lack of cortical diagnostic findings in the anterior temporal lobe.

### 3.2 Visual assessment

**Fig. 2:**
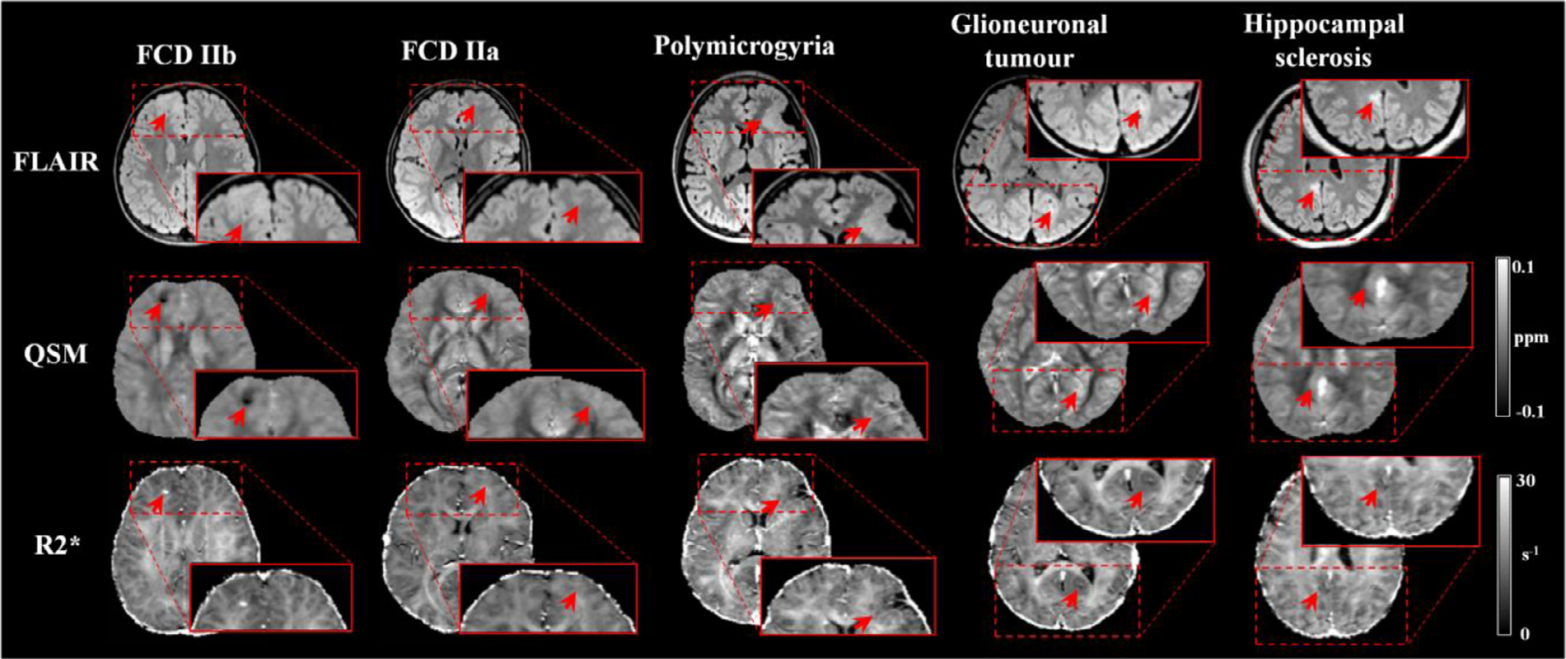
Examples of QSM and R2* maps obtained in the clinical setting for patients with FCDIIb, FCDIIa, polymicrogyria, Glioneuronal tumour and hippocampal sclerosis (HS). The red arrow points toward the lesion identified by radiologists. For the HS patient, the arrow highlights an abnormal region in the posterior cingulate gyrus as identified on the radiological report.

Examples of patients’ with visible alterations on QSM (all patients had a mean score of lesion visibility equal or above 3), spatially concordant with changes visible in FLAIR images, are shown on Fig. 2 with comparison to R2* maps.

Compared to the best visualisation achieved in either FLAIR or T1w, the lesions conspicuity was visually assessed as being better/equal/worse on the QSM in respectively 2/3/35 individuals and on the R2* in 3/3/34.

The lesion conspicuity was scored as being significantly different across image types (*p*<0.001). The post-hoc multiple comparison test applied to the Friedman ranking test showed that the mean ranking of FLAIR and T1w was not significantly different, while the mean ranking of R2* and QSM was significantly reduced compared to FLAIR images (*p*<0.05).

### 3.3 QSM, R2* maps and SRXRF elemental analysis

The SRXRF analysis was carried out on samples from two FCD IIb patients, a patient with a glioneuronal tumour, and on the temporal lobe tissue resected from of a HS patient. This patient had partial hippocampal and standard temporal lobe resection involving the anterior temporal lobe; however the latter tissue did not show specific diagnostic changes after the histological examination. Therefore, we used the portion of the anterior temporal lobe as control tissue for the experiment.

**Fig. 3:**
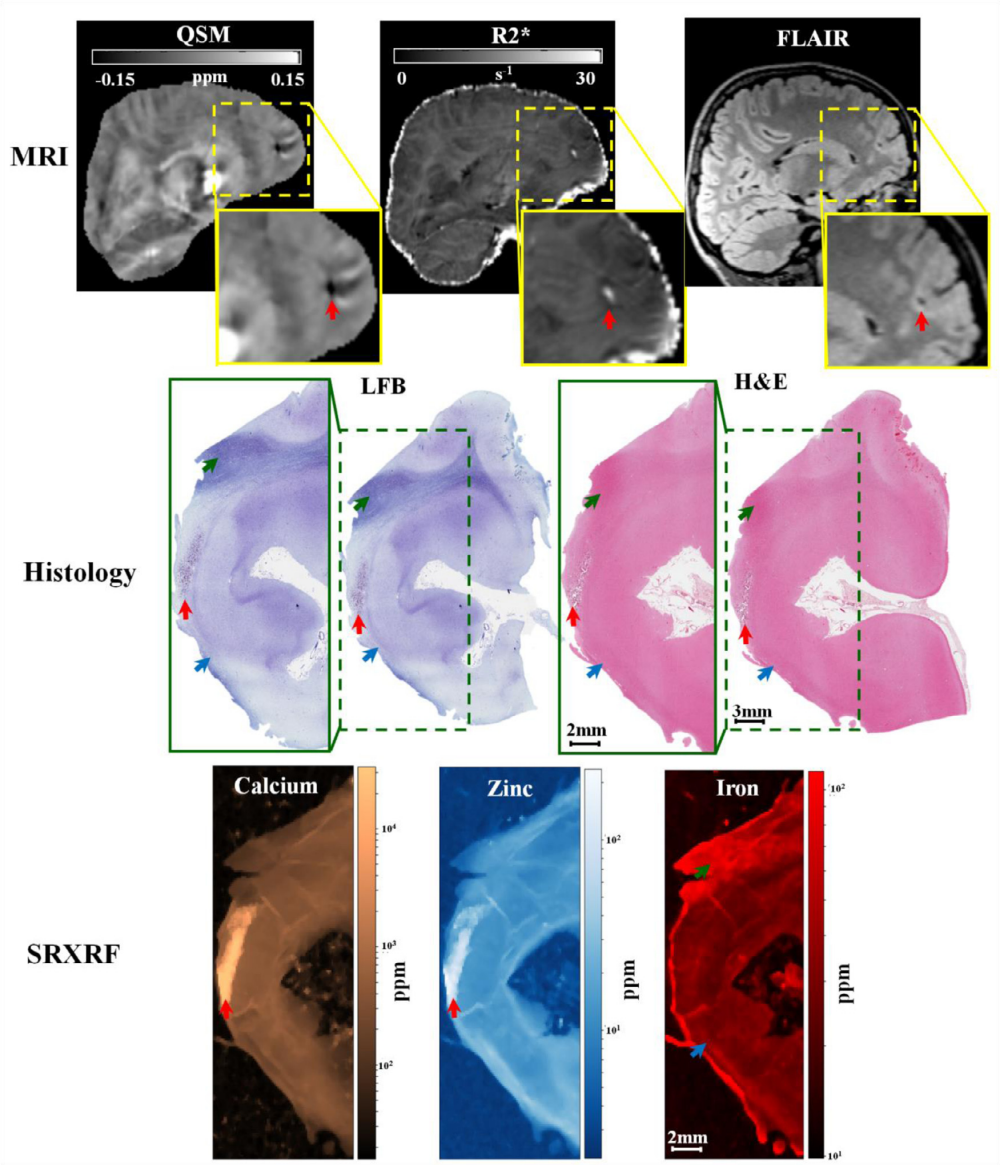
Example 1 of FCDIIb lesion analysed using MRI, histology and synchrotron radiation X-ray fluorescence (SRXRF). This case corresponds to FCDIIb case reported on Fig. 2. In the upper row, the FCD lesion is delineated by hypo-intensity by QSM (↑), hyper-intensity by R2* (↑), and cortical thickening by FLAIR (↑). The middle row shows histological luxol fast blue (LFB) and haemotoxylin-eosin (H&E) staining of sections from the resected tissue. LFB identifies an area with low myelin content (↑) and an area with normal appearing myelin (↑) in the white matter, while H&E stains mineral deposits (↑) dark purple. The bottom row shows the SRXRF elemental maps for calcium, zinc and iron. Increased calcium and zinc (↑) are present in the area corresponding to mineral precipitates as assessed by H&E, and to the hypo-intense region (↑) on QSM. Iron content in the SRXRF map is lower in the subcortical white matter underneath the sulcus (↑) compared to that in normal appearing white matter (↑).

For the first FCDIIb patient (patient 8), we observed increased calcium in the lesion (15 × 10^3^±5 × 10^3^ppm), compared to neighbouring normal tissue (335±52 ppm). We found increased zinc in the lesions (113.7 ± 39.2 ppm), compared to neighbouring tissue (13.9 ± 0.9 ppm). The region exhibiting increased zinc and calcium in the tissue was compatible with the findings of low χ and high R2* values in the lesion (Fig. 3). Moreover, in the subcortical white matter belonging to the lesion, there was decreased iron (Fe=16±1 ppm), compared to normal white matter (Fe=36±10 ppm). A visual inspection of the LFB staining showed that the areas exhibiting low myelin were consistent with those with low iron content (Fig. 3).

**Fig. 4:**
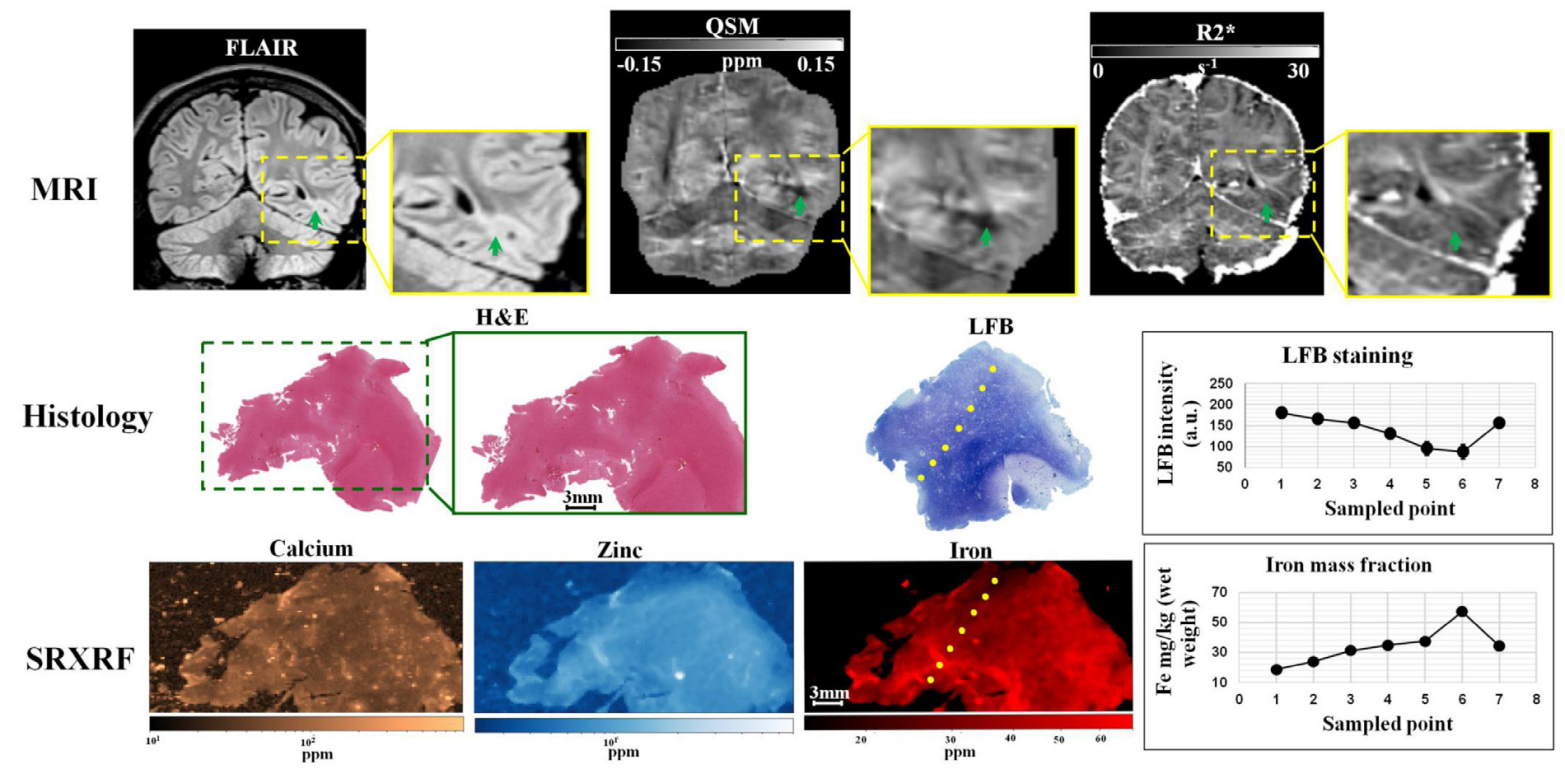
Example 2 of FCDIIb lesion analysed using MRI, histology and synchrotron radiation X-ray fluorescence (SRXRF). In the upper row, the green arrow points toward the FCD lesion delineated by cortical thickening on FLAIR and by R2*, and hypo-intensity on QSM. The middle row shows histological haemotoxylin-eosin (H&E) and luxol fast blue (LFB) staining performed on sections of resected tissue. The LFB identifies an area with low myelin content at the top of the dotted line. The LFB intensity profile along the dotted line is plotted on the graph on the right side of the middle row. The bottom row shows the SRXRF elemental maps for calcium, zinc and iron. Low iron content is observed at the top of the dotted line and iron content along the dotted line is plotted on the graph on the right side of the bottom row. The dotted line is arbitrary drawn, however looking along this line, the LFB staining intensity and the iron content graphs indicate that myelin increase (decrease in LFB intensity) corresponds to higher iron content.

For the second FCDIIb patient (patient 15) we found decreased iron in the FCD lesion (19±2 ppm) with respect to the neighbouring tissue (40±2 ppm) (Fig. 4). The zinc content in the lesion was 5.2 ± 0.6 ppm; while in the neighbouring tissue 9 ± 2.6 ppm. Calcium appeared to be similar in the lesion and surrounding tissues. A visual inspection of the LFB staining showed that the areas exhibiting low myelin where the ones with low iron content (Fig. 4).

**Fig. 5:**
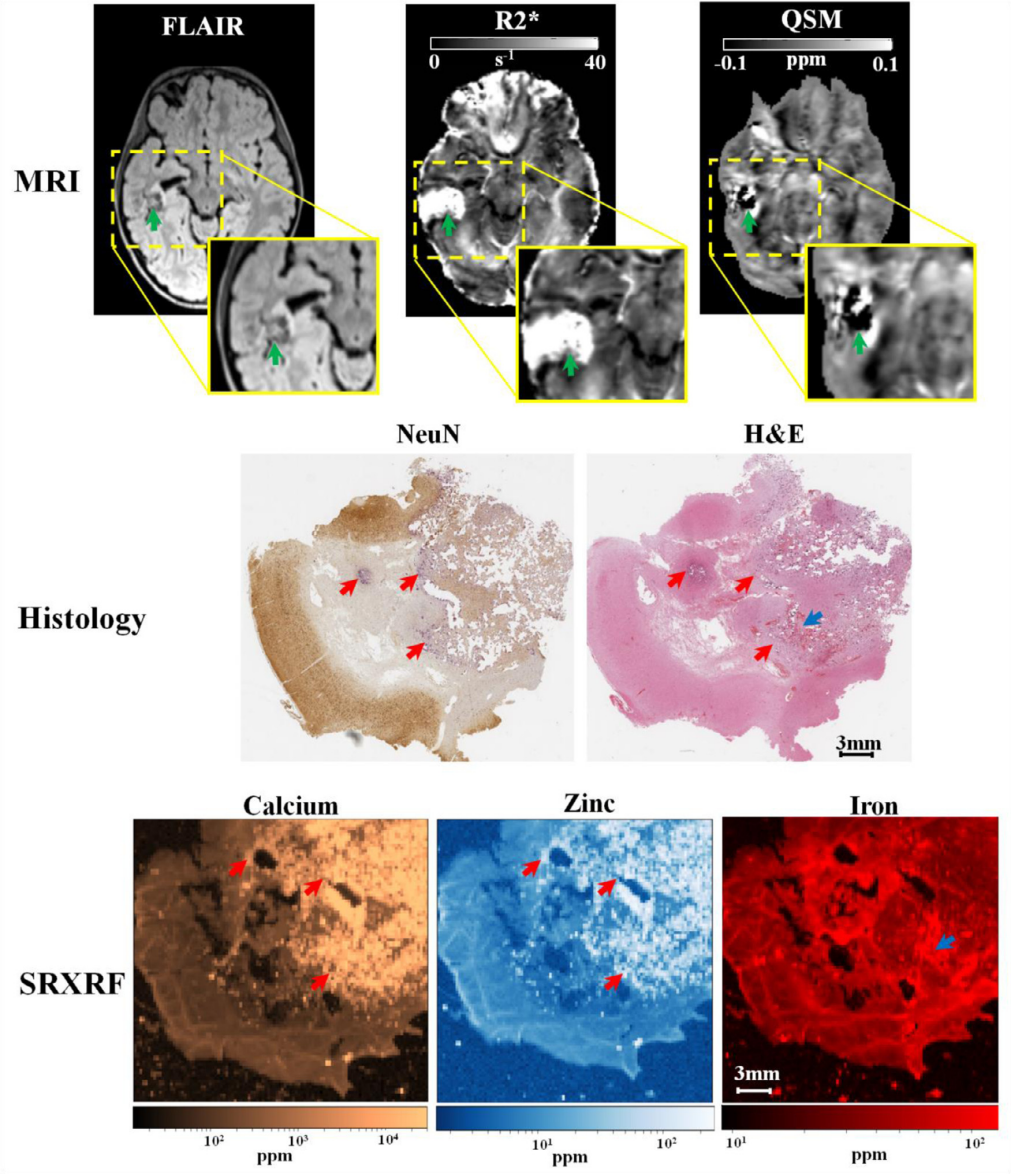
Example of glioneuronal tumour analysed using MRI, histology and synchrotron radiation X-ray fluorescence (SRXRF). In the upper row, the green arrow points toward the lesion delineated by abnormal intensity on FLAIR hyper-intensity on R2* and hypo-intensity on QSM. The middle row shows optical micrographs of sections from the resected tissue stained with neuraminidase (NeuN) or haemotoxylin-eosin (H&E) section. Both NeuN and H&E identify areas with mineral deposits (↑) stained in dark purple. The bottom row shows the SRXRF elemental maps for calcium, zinc and iron. Increased calcium and zinc (↑) are present in those areas identified to be have mineral precipitates by NeuN and H&E staining, and are hypo-intense region on QSM. High iron content is present in the region highlighted by the blue arrow (↑) which appears to be blood by H&E staining (↑).

Additionally, we performed SRXRF analysis on the tumour region from patient 22 diagnosed with glioneuronal tumour. We found: increased calcium (11 × 10^3^±7 × 10^2^ppm) within the tumour compared to adjacent normal appearing grey matter (200±50 ppm), increased zinc (11 × 10^2^±7 × 10 ppm) within the tumour compared to normal appearing grey matter (70±50 ppm), increased iron (120±40 ppm) in areas where small blood vessels were present compared to normal appearing grey matter (50±30 ppm) (Fig. 5). The areas with increased zinc and calcium on the SRXRF maps were consistent with regions showing low χ and high R2* values on the MRI maps.

For the control tissue without histopathological abnormalities resected from the anterior temporal lobe, we also quantified calcium, zinc, and iron in both the grey and white matter (Ca WM: 63.3 ± 7.2 ppm Ca GM: 113.2 ± 59.5 ppm; Zn WM: 7.5 ± 0.9 ppm, Zn GM: 12.4 ± 2.8 ppm; Fe WM: 45.8 ± 5 ppm, Fe GM: GM: 30.1 ± 1.5 ppm).

### 3.4 Quantitative lesion profiling

**Fig. 6:**
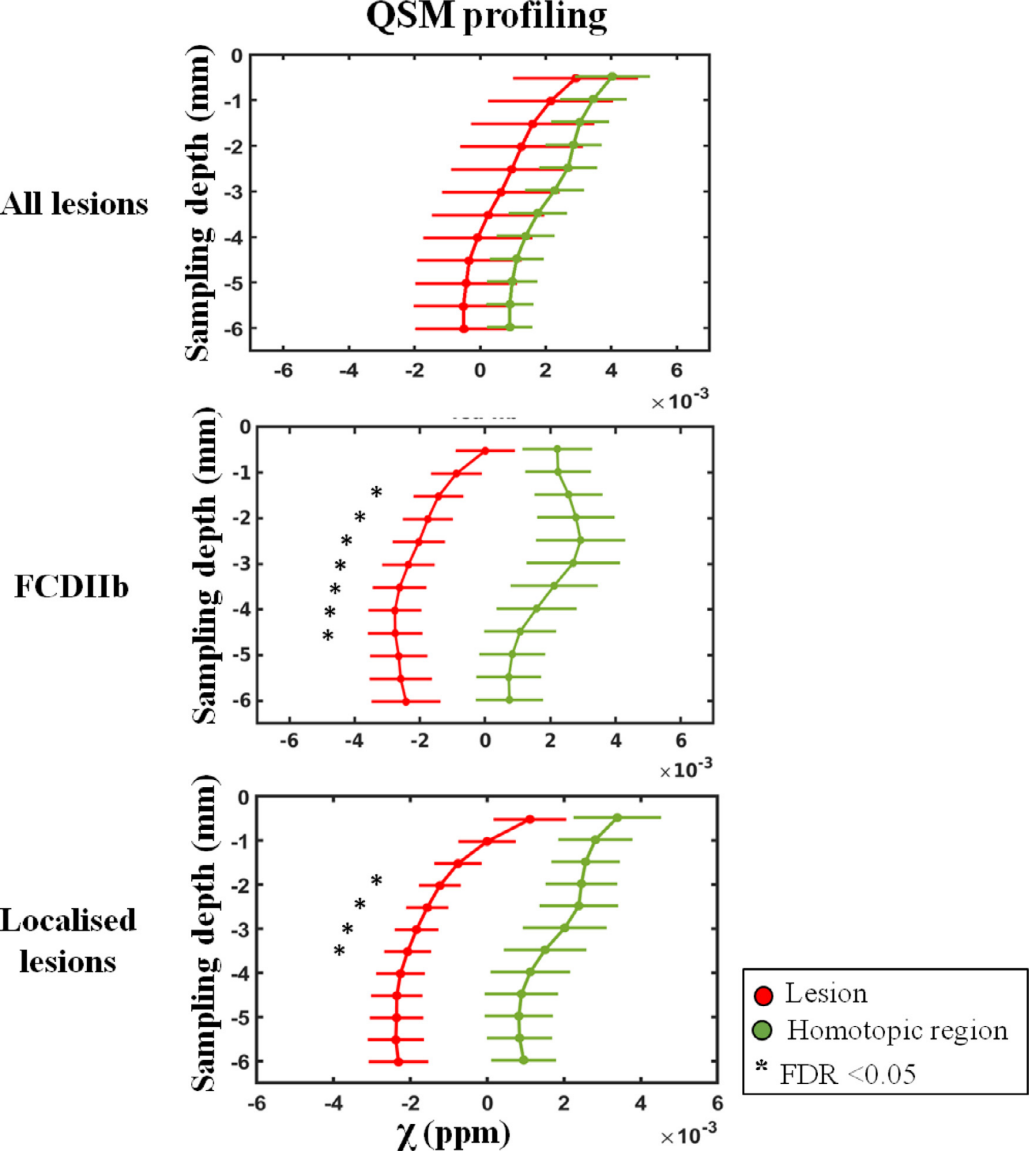
MRI profiling of FCD lesions and homotopic regions. The plots show QSM-based profiling obtained for lesion (red) and homotopic regions (green) for the whole cohort of patients, histology confirmed FCDIIb and well-localised lesions (including histology confirmed lesions and suspected FCD lesions with EEG-based seizure semiology concordant with MRI-based radiology report). The sampling depth is reported as a distance from the pial surface (0 mm). Error bars correspond to standard error of the mean. (*) identifies significant profile changes between lesion and homotopic area at false discovery rate (FDR) <0.05.

No significant χ differences were found across the whole patient cohort between the lesion profiles and homotopic region ones (Fig. 6). However, when restricting the analysis to well-localised lesions we observed decreased χ values across cortical depths with significant differences at 2–3 mm with respect to the homologous regions (p_FDR_<0.05). Similar results were obtained for the sub-group with histologically confirmed FCDIIb lesions, as shown on Fig. 6. We did not observe any significant R2* differences between the lesions and homotopic regions in any of the patients groups.

### 3.5 Age effect in deep brain nuclei

We found positive partial linear correlation of both age and disease duration with χ and R2* values separately in all deep brain nuclei (see Table 2). The nuclei with the highest correlation between age and χ where the caudate, globus pallidus and substantia nigra (correlation >0.65), while the regions with the highest correlation between age and R2* where the caudate and globus pallidus (correlation >0.61) (see Table 2 and Figure 7 in Supplementary material).

**Table 2:** Partial correlation coefficients of the mean susceptibility (χ) and R2* with age and disease duration.

Map	Deep brain region	Correlation coefficient – left side		Correlation coefficient – right side	
		Age	Disease duration	Age	Disease duration
χ	Caudate	0.66	0.13	0.67	0.15
	Putamen	0.55	0.37	0.46	0.33
	Pallidum	0.79	0.40	0.78	0.43
	Thalamus	0.12	0.40	0.12	0.40
	Substantia nigra	0.65	0.34	0.67	0.20
	STN	0.40	0.13	0.53	0.11
	Red nucleus	0.37	0.21	0.50	0.31
	Cerebellar dentate	0.29	0.16	0.28	0.17

R2*	Caudate	0.60	0.25	0.61	0.23
	Putamen	0.37	0.17	0.36	0.15
	Pallidum	0.64	0.32	0.65	0.30
	Thalamus	0.33	0.13	0.34	0.10
	Substantia nigra	0.26	0.14	0.27	0.12
	STN	0.33	0.16	0.32	0.18
	Red nucleus	0.22	0.11	0.23	0.09
	Cerebellar dentate	0.36	0.10	0.37	0.15

The correlation coefficients were estimated separately for each deep brain structure and each brain side for the whole group of patients. STN = sub-thalamic nucleus.

To determine if age and disease related changes in iron levels in deep brain nuclei could be detected by alterations in χ and R2* values, a multiple linear regression model was used. There was linearity of the dependent variables and the χ, R2* values, as assessed by partial regression plots and a plot of studentized residuals against the predicted values. There was no evidence of multi-collinearity between independent variables, as evidenced by no tolerance values less than 0.2. The assumption of normality was met, as assessed by a Q-Q Plot.

The multiple regression model significantly (*p*<0.05) predicted the mean χ in the caudate (left and right), putamen (left and right), pallidum (left and right), substantia nigra (left and right), sub-thalamic nucleus (left and right), red nucleus (left and right), as reported on Table 3 in Supplementary material. Age was a significant (*p*<0.05) predictor for the caudate, pallidum and substantia nigra (Table 3 in Supplementary material). Disease duration was also a significant predictor for the putamen, pallidum and substantia nigra (Table 3 in Supplementary material). The regression models applied respectively within the well-localised and FCDIIb patients’ sub-groups together with controls, showed similar age and disease correlation in the same nuclei identified in the whole group analysis.

There was not a statistically significant effect of disease on the relationship between age and the mean χ values within any of the structures as evidenced by increase in total variation explained (*p*>0.100) as reported on Table 3 in Supplementary material. Similar results were obtained for the model applied to the patients’ sub-groups.

The multiple regression model significantly (*p*<0.05) predicted the mean R2* in the caudate (left and right), putamen (left and right) and pallidum (left and right), as reported on Table 3 in Supplementary material. Age was a significant (*p*>0.05) predictor for the R2* values in the caudate and pallidum (Table 3 in Supplementary material). The disease duration was not a significant predictor for the mean R2* value in any of the deep brain nuclei. The regression models applied on the healthy controls and the patients’ sub-groups showed significant age correlation in the same nuclei identified in the whole group analysis.

There was not a statistically significant effect of disease on the relationship between age and the mean R2* values within any of the structures as evidenced by increase in total variation explained (*p*>0.100) as reported on Table 3 in Supplementary material. Similar results were obtained within the FCDIIb and well-localised patient sub-groups.

## 4 Discussion

To our knowledge, this is the first study using QSM to characterise epileptogenic lesions in humans. QSM was evaluated in a paediatric population of forty patients with drug-resistant focal epilepsy.

Our primary hypothesis was that *focal* reductions in χ will be found in lesions driven primarily by alterations in their iron and calcium content. Consistent with our hypothesis, we demonstrated significant local χ decrease in MCD lesions, which was associated with reduced iron and myelin in several patients, and increased calcium and zinc in some cases. Measurements of χ across cortical depth in groups with well-localised and confirmed FCDIIb lesions both showed a reduction in χ at 2–3 mm compared with homologous cortical regions. In two cases, the reduction in χ was associated with lower iron content that was linked with a pathological reduction in myelin observed by SRXRF and histopathological analyses, respectively.

Our second hypothesis was that regional changes in brain iron in the deep grey matter nuclei might be present in children with poorly controlled focal epilepsy compared to healthy control subjects. We found a positive correlation between age and both χ and R2* values in deep brain nuclei, consistent with previous reports (Hallgren and Sourander, 1958; Ordidge et al., 1994; Yao et al., 2009; Ashraf et al., 2018; Ghassaban et al., 2019), and there was also a positive correlation with disease duration. However there was not a significant interaction of disease on the relationship between age and the χ and R2* values within any of the structures.

### 4.1 Alterations in cortical structure detected by QSM

The quantitative lesion profiling analysis showed consistent changes in χ for well-localised lesions and this was also seen in the FCDIIb sub-group. The analysis of χ with cortical depth (Fig. 6) showed that lesions exhibited negative χ values across different sampling depths. In order to shed light on the tissue mineral substrate underlying these negative χ values, SRXRF was performed to identify and quantify alterations in multi-elemental concentrations. Increased calcium and zinc were observed in one FCDIIb sample. Both substances are diamagnetic and their presence is in agreement with hypo-intense QSM (and hyper-intense R2*) observed in the same sample. Previous studies on an animal model for epilepsy, revealed the presence of localized calcium deposits in diamagnetic regions delineated on QSM (Aggarwal et al., 2018; Lafreniere et al., 1992), however the alterations in zinc observed here, have not been previously described.

Dystrophic calcifications have been observed in a spectrum of neurological disorders, and are associated with abnormal mineral accumulation in areas of degenerated or necrotic tissue (Casanova and Araque, 2003). In two patients, one with FCD IIb and a glioneuronal tumour strongly visible reduced χ changes were associated with high levels of calcium accumulation and therefore, may indicate lesions that have a high degree of tissue damage.

Zinc is involved in many biochemical processes and is a crucial micronutrient necessary for normal brain functions (Mocchegiani et al., 2005; Strong et al., 2020). The mechanism by which zinc is taken up in the brain parenchyma cells is poorly understood (Takeda, 2000). High zinc levels have also been related to excitotoxicity in a variety of conditions, including ischemia, brain trauma and epilepsy (Pei et al., 1983; Sensi et al., 2011, 2009) and aggregation of β-amyloid protein, the main component of the senile plaques typically observed in Alzheimer's disease brains (Frederickson et al., 2000). Zinc accumulation can cause both neuronal and glial death in vitro and in vivo (Aizenman et al., 2000; Hwang et al., 2008).

Although the precise mechanisms of brain tissue mineralization are not currently understood, localized increase in intracellular calcium and zinc concentration have been associated with apoptotic or necrotic cell death and inflammatory processes (Casanova and Araque, 2003; Liu et al., 2015b; Sensi et al., 2009).

Moreover, we found that, while the lesions showed uniform negative χ across sampling depths, homotopic regions had positive χ that peaked between 2 and 3.5 mm depth. This results are in agreement with high resolution QSM maps acquired at 7T showing that deeper layers of the cortex present increased χ values due to the presence of an iron-rich layer (Fukunaga et al., 2010; Marques et al., 2010). This conclusion was based on the application of high field susceptibility maps together with histology and x-ray fluorescence and on post-mortem brains (Deistung et al., 2013a; Fukunaga et al., 2010; Stüber et al., 2014). Our findings at 3T of increased χ values in non-lesional cortex, peaking at ~2.5 mm are entirely consistent with a high iron layer corresponding to underlying myeloarchitecture. Although the myeloarchitecture changes across cortical regions, there is a consistent pattern of high myelination of deep cortical layers compared to superficial ones (Timmler and Simons, 2019). This finding is concordant with the few QSM studies that have evaluated whether iron is co-localised with myelin in deep cortical layers in healthy subjects (Deistung et al., 2013b; Stüber et al., 2014).

This pattern of increased χ values at 2–3.5 mm depth was absent in lesions both in the well-localised, and the histologically confirmed FCDIIb sub-group. Some FCD lesions sub-types have disrupted radial and tangential laminar structure of the cortex (Blumcke et al., 2017), that would be expected to alter typical myeloarchitecture, particularly in deep cortical layers and at the grey-white matter interface. Further, FCDIIb is associated with demyelination (Blumcke et al., 2017), consistent with the T2 FLAIR hyperintensity often seen radiologically (Blümcke et al., 2011). The absence of this peak in χ at 2–3.5 mm cortical depth represents a potential signature of cortical dysplasia that might be useful for its identification and classification particularly in the context of ongoing QSM image quality development.

MCD lesions also have alterations in cytoarchitecture, with FCDIIb having dysmorphic neurons and balloon cells. The alterations in mineral content that might be associated with these abnormalities are largely unknown. SRXRF analysis of tissue samples of the both FCDIIb patients showed decreased iron in the brain tissue from the lesion. The iron decrease was spatially concordant with the myelin depletion highlighted by histological staining (Figs. 3, 4), which could potentially explain both the overall decrease in χ and the lack of the peak in χ values in the lesional profile. The lack of a decrease in iron in the control tissue supports this finding. Despite the decrease of paramagnetic substances, such as iron, and diamagnetic ones, such as myelin, have opposite effect on the χ values (Langkammer et al., 2018), it has been shown by previous studies that iron and myelin do not co-localize at constant proportions throughout the brain, instead intracortical fibres have a disproportionately higher iron than myelin content (Duyn et al., 2007; Fukunaga et al., 2010).

However, the changes in χ values we report, while associated with changes in tissue composition were not designed to establish the exact biophysical relationship or causative drivers of QSM changes in terms of tissue composition. Additional studies on the quantification of iron and myelin type and concentration, and their correlation with QSM signal would improve our understanding of the specific basis of alterations in χ values.

We found that when including subjects with poorly localised focal epilepsy (for example discrepant seizure semiology and MRI lesion location) we did not find significant χ profile differences between FCD lesions and homotopic regions (Fig. 6: top row). This might suggest that the reduced χ values are specific to the true epileptogenic lesion. The results found here motivate future studies, designed and powered to evaluate the potential to use this signature of MRI χ changes for lesion detection and stratification.

SRXRF elemental mapping on the specimen with glioneuronal tumour revealed increased calcium and zinc in tumour tissue. The presence of these diamagnetic substances is consistent with hypo-intensity on QSM and hyper-intensity on R2*. The pathophysiology underlying the mineralization of this brain tumour is not currently understood (Ciraci et al., 2017; Deistung et al., 2013b; Eskreis-Winkler et al., 2017). The tumour specimen also showed the presence of increased iron that could be due to bleeding. Notably, data was obtained from only one patient and more studies are required to determine trace elemental concentrations in such brain tumours (Cilliers et al., 2020).

### 4.2 Age effect in deep brain nuclei

In agreement with previous studies carried out in paediatric population (Darki et al., 2016; Li et al., 2014; Peterson et al., 2019; Zhang et al., 2018), a significant linear correlation was observed between age and χ measured in the caudate, globus pallidus, substantia nigra, sub-thalamic nucleus and red nucleus for the whole cohort of patients and healthy controls. Moreover, we found a significant linear dependency between age and the mean R2* in the caudate and globus pallidus. These nuclei are known to have the greatest age-related iron accumulation of all the deep brain structures investigated in this study, leading to increased χ and R2* values, (Li et al., 2014; Zhang et al., 2018).

Other structures such as the putamen, thalamus and cerebellar dentate are also known to accumulate iron, however at a slower rate or resulting in a lower mean susceptibility value, and, therefore, correlations between χ, R2* and age in these regions did not reach statistical significance given the relatively small group size (*n* = 40) and age range (2–21). It should also be noted that the spatial location of focal epilepsy varied within this cohort and, therefore, more spatially localised changes in regions such as the thalamus could be present in larger more homogenous cohorts reflecting volumetric and functional connectivity studies (Centeno et al., 2014; He et al., 2017; Perani et al., 2018). We found potential evidence of increased brain iron deposition due to epilepsy duration that could reflect accelerated brain-ageing in this paediatric cohort. In this patient group, seizure onset is typically early in life and therefore age and duration of epilepsy are highly correlated. Never-the-less our model did suggest that duration of epilepsy did explain significant additional differences in several deep brain nuclei. This interesting finding should be confirmed in a group with a wider range of onsets and ages.

### 4.3 Visual assessment

Based on the multiple comparison statistical tests, the QSM and R2* maps provided worse FCD lesion conspicuity compared to FLAIR and T1w images. At the individual level, the lesion contrast on QSM and R2* was enhanced in respectively 5% and 7.5% of patients; therefore, these maps are most likely to provide information in combination with FLAIR to detect and characterise MCD. QSM provided very high contrast in a small but significant number of patients which was related to calcium and zinc increases. The sensitivity to calcification may be useful for targeting in cases with widespread abnormalities such as tuberous sclerosis. Future studies are needed to validate the radiological value of maps in larger cohorts including in MRI-negative patients.

### 4.4 Limitations and outlook

The clinical trend is to perform surgical resection on patients with clearly defined epileptogenic regions and seizures that affect both quality of life and development. It is therefore crucial to non-invasively characterise focal lesions in order to improve surgical planning, seizure freedom and neuro-developmental outcome (Adler et al., 2017a; Choi et al., 2018). This study is the first investigation using QSM in epileptic lesions to reveal alterations in iron content that appear to be related to myeloarchitecture associated with MCD. An observed MRI abnormalities within the epileptogenic zone (presumed *n* = 4, confirmed *n* = 21) was reduced χ and the absence of a peak in χ at a cortical depth of ~2.5 mm. The consistency of χ changes across different cortical regions in FCDIIb lesions indicates that these maps could be useful for the characterisation of those lesions and could be integrated into algorithms for automated lesion detection (Adler et al., 2017a; Lorio et al., 2020). Future studies are needed to validate cortical QSM profiles for automated lesion detection in larger cohorts.

The use of the pial surface as a reference for the sampling of χ and R2* values could be inaccurate especially at lower depths, such as 5–6 mm, where intricate folding patterns are present. However significant χ changes were observed at sampling depth closer to the pial (1.5–4.5 mm depth for the FCDIIb lesions, and at 2–3.5 mm depth for localised lesions), that are less affected by inaccuracies of surface reconstruction. The iterative erosion applied during the brain masking for the QSM estimation could potentially remove voxels on the brain surface with high calcium or zinc content generating large phase values on the phase maps. Despite this possible limitation we were able to analyse the susceptibility maps in regions with high mineral content few millimetres from the pial surfaces.

In 5% of patients, the lesions were visually more conspicuous on QSM with respect to FLAIR and T1w images. Some lesions with negative χ were identified and further analysed with SRXRF in two patients with FCDIIb and one patient with a glioneuronal tumour showing calcium and also zinc increases. The clinical relevance of these changes in tissue composition is not clear. However, in tuberous sclerosis, which has histopathological similarities to FCDIIb, calcification has been reported to be dynamic and associated with epileptogenicity (Gallagher et al., 2010). The high zinc levels associated with regions of reduced QSM may be related to neurotoxicity and gliotic changes. The presence of extracellular Zn^2+^ is known to alter neuronal excitability and therefore facilitate and modulate epileptiform activity in the hippocampus (Carver et al., 2016; Coulter, 2000), while its role in epileptogenic cortical abnormalities remains largely unknown.

Like calcium, zinc is a diamagnetic element that can drive relaxivity and susceptibility signal changes on MR images (Byrne et al., 2014; Dhulipala et al., 2018). Further studies are needed to directly correlate the shift of susceptibility values with the amount of mineral content.

We provided preliminary evidence of an association between χ value changes and elemental alterations measured in a modest sub-group. However, this study was not designed to disambiguate the causative drivers of changes in magnetic susceptibility estimated by QSM in terms of particular elemental concentrations. While we demonstrated that reductions in χ were associated with a combination of iron and myelin reductions with calcium and zinc increases in the specimens available, it is plausible that different combination of changes in tissue composition could also result in QSM reductions (Stüber et al., 2016; Wiggermann et al., 2017; Wisnieff et al., 2015).

Owing to the limitations both practical and technical, exact coregistration between imaging findings and SRXRF was not possible. Due to the qualitative nature of this spatial comparison, some caution is needed in the interpretation of the exact relationship between elemental changes and QSM values, particularly at smaller spatial scales. Future studies including imaging of resected tissue prior to neuropathological assessment would be advantageous to confirm changes and make a spatial comparison between the findings. This can be difficult because of the variable nature of FCD surgery in terms of spatial location and surgical approach that can limit the availability of tissue particularly at resection margins.

QSM is a rapidly developing field of MRI with increasingly sophisticated acquisition and, in particular, complex post-processing algorithms. The magnetic susceptibility is estimated from the MRI signal phase, which is affected by the contributions from local microscopic tissue composition and macroscopic background fields which are removed via post-processing. QSM is also sensitive to acquisition parameters (particularly TE) (Biondetti et al., 2020) and tissue orientation with respect to the main magnetic field (Schweser et al., 2011; Wharton and Bowtell, 2015, 2012). Despite these limitations we found consistent differences in susceptibility values in the cortex. This is a unique finding because QSM has commonly been applied to iron-rich deep brain regions (where we also found age-related susceptibility increases). In this study QSM was performed at 3T, demonstrating clinical relevance, however phase-based measurements, such as QSM, benefit from increases in contrast and resolution at 7T, therefore this study strongly motivates further application of QSM to study patients with epilepsy at 7T.

## CRediT authorship contribution statement

**Sara Lorio:** Methodology, Software, Validation, Formal analysis, Investigation, Data curation, Writing - original draft. **Jan Sedlacik:** . **Po-Wah So:** Resources, Validation, Investigation, Formal analysis, Writing - review & editing, Visualization. **Harold G. Parkes:** Resources, Investigation. **Roxana Gunny:** Formal analysis, Investigation. **Ulrike Löbel:** Formal analysis, Investigation. **Yao-Feng Li:** Formal analysis, Investigation, Data curation. **Olumide Ogunbiyi:** Formal analysis, Investigation. **Talisa Mistry:** Formal analysis, Investigation. **Emma Dixon:** Methodology, Software. **Sophie Adler:** Methodology, Software, Writing - review & editing. **J. Helen Cross:** Resources, Funding acquisition, Writing - review & editing. **Torsten Baldeweg:** Resources, Writing - review & editing. **Thomas S. Jacques:** Writing - review & editing, Resources, Supervision, Data curation. **Karin Shmueli:** Methodology, Software, Validation, Writing - review & editing. **David W Carmichael:** Conceptualization, Methodology, Formal analysis, Investigation, Supervision, Project administration, Funding acquisition, Writing - original draft, Writing - review & editing.

## Declaration of Competing Interest

None of the authors has any conflict of interest to disclose.

## Appendix Supplementary materials

Image, application 1
