# Supplementary material for: Quantitative MRI susceptibility mapping reveals cortical signatures of changes in iron, calcium and zinc in malformations of cortical development in children with drug-resistant epilepsy
Source: Neuroimage. 2021 Sep;238:118102. doi: 10.1016/j.neuroimage.2021.118102 (PMC8350142; doi:10.1016/j.neuroimage.2021.118102)
Supplement: Supplementary file 1 [file mmc1.docx]

**Supplementary material**

**Histology staining**

Formalin-Fixed-Paraffin-Embedded (FFPE) tissue blocks were obtained from Great Ormond Street Hospital (GOSH) for Haematoxylin and Eosin staining (H&E), Luxol Fast Blue (LFB), Neuropathology special stain, Immunohistochemistry (IHC) and slide scanning.

H&E staining

Sections were cut at 5μm thickness and stained on Leica ST5010/CV5030 automated workstation. The automated machine incubated the sections in 2 changes of xylene (Genta Medical UN1307), followed by rehydration in graded alcohols (Genta Medical UN1170) and a wash step in distilled water. Next, the sections were stained with Harris Haematoxylin (Leica Surgipath; Ref 3801560E) for 6 minutes 30 seconds exactly, then washed in distilled water and briefly differentiated in 1% acid alcohol for 10 seconds to remove excess haematoxylin. Subsequently, the sections were blued in distilled water followed by counterstaining in 1% Eosin (Leica Surgipath; Ref 3801590E) for approximately 4 minutes 30 seconds. Afterward, the sections were washed in distilled water, dehydrated in graded alcohols and cleared in 2 changes of xylene in preparation for mounting. The sections were mounted with Pertex (Histolab; Ref 00801) mounting media for light microscopy.

Luxol Fast Blue (LFB)

Sections were cut at 14 μm on super frost adhesive slides, baked overnight in 37 °C oven and in 60°C oven for 1 hour the following morning. Slides were de-waxed in xylene, rehydrated in graded alcohols and placed in a staining dish containing filtered luxol fast blue in acidified methanol (Atom Scientific; Ref RRSP248-G, Lot 116149). The slides were immediately transferred into a water bath pre-heated to 60°C and left to incubate for 2 hours. Afterward, the sections were briefly rinsed with denatured 70% ethanol (Atom Scientific; Ref RRSP242-G, Lot 116642) and distilled water before differentiating in 0.05% lithium carbonate solution (Atom Scientific; Ref RRSP252-G, Lot 113742) until the grey and white matter can be distinguished. Subsequently, the slides were rinsed in 95% alcohol and water followed by counterstaining with cresyl violet 0.5% aqueous solution (Atom Scientific; Ref RRSP251-G, Lot 116921) for 10-12 minutes. Briefly, the sections were washed in water and differentiated in a solution of 1% acetic acid in 95% denatured ethanol (Atom Scientific; Ref RRSP260-G, Lot 113792) for up to 4 seconds. The sections were rapidly dehydrated through graded alcohols, cleared in 2 changes of xylene and mounted with pertex mounting media as previously described.

**Immunohistochemistry (IHC)**

IHC staining was performed on a Leica Bond-Max auto-stainer (Leica Bio systems, Melbourne, Australia) and the Leica Bond Polymer Refine Detection kit (Ref. DS9800) was used to visualise bound antibody. Neuron specific protein expression was detected using anti- NeuN (clone A60), mouse monoclonal primary antibody catalogue no. MAB377 (Merck Millipore, Billerica, MA, USA), with an optimized dilution of 1:500. To validate IHC staining, a surgical brain tissue with known expression of the target protein was used as positive control and Tissue Micro Array (TMA) was used as negative control by omitting the application of the primary antibody. On the Leica Bond-Max machine, the sections were de-waxed with xylene and rehydrated with 99% Industrial Denatured Alcohol (IDA). Antigen retrieval was achieved by Heat Induced Epitope Retrieval (HIER), Leica Epitope Retrieval protocol 2 (ER2) for 30 minutes, pH 9, Bond-max protocol F. Peroxidase block was applied for 5 minutes with Bond polymer refine kit (Ref. DS9800) followed by the application of the primary antibody for 1 hour. The sections were subsequently incubated with post primary (Bond polymer refine kit; Ref. DS9800) for 8 minutes and HRP labelled polymer (Bond polymer refine kit; Ref. DS9800). The application of 3, 3-Diaminobenzidine (DAB) (Bond polymer refine kit; Ref. DS9800) chromogen solution to the sections forms a brown precipitate at positively expressed antigen sites. The tissue sections were counterstained with haematoxylin (Bond polymer refine kit; Ref. DS9800) and mounted for light microscopy as previously described. The test tissues were cut at 5μm & controls at 3 μm thickness and mounted on a Leica Surgipath X-tra adhesive slides.

Slide scanning

The stained sections were scanned on the Leica Aperio CS2 Scanner (S/N 5872) at 40x objective.

| Map | Deep brain region | Model predictors p-value | | | R^2^ | Adj R^2^ | F-value (degree of freedom) | p-value | Interaction disease – age | | |
| --- | --- | --- | --- | --- | --- | --- | --- | --- | --- | --- | --- |
|  |  | **Age** | **Disease presence** | **Disease duration** |  |  |  |  | **R^2^ change** | **F-value change (degree of freedom)** | **p-value** |
| χ | Caudate L | 0.001* | 0.910 | 0.579 | 0.321 | 0.283 | 8.355 (3,53) | <0.001* | 0.070 | 5.96 (1,52) | 0.185 |
|  | Caudate R | 0.007* | 0.402 | 0.101 | 0.279 | 0.211 | 6.007 (3,53) | 0.001* | 0.026 | 1.845 (1,52) | 0.180 |
|  | Putamen L | 0.097 | 0.323 | 0.009* | 0.284 | 0.228 | 6.977 (3,53) | <0.001* | <0.005 | 0.035 (1,52) | 0.853 |
|  | Putamen R | 0.084 | 0.162 | 0.008* | 0.319 | 0.267 | 7.932 (3,53) | <0.001* | 0.009 | 0.724 (1,52) | 0.399 |
|  | Pallidum L | <0.001* | 0.001* | <0.001* | 0.669 | 0.65 | 35.636 (3,53) | <0.001* | 0.001 | 0.200 (1,52) | 0.657 |
|  | Pallidum R | <0.001* | 0.001* | <0.001* | 0.661 | 0.642 | 34.465 (3,53) | <0.001* | 0.005 | 0.794 (1,52) | 0.377 |
|  | Thalamus L | 0.889 | 0.285 | 0.304 | 0.188 | 0.142 | 4.097 (3,53) | 0.110 | <0.005 | 0.026 (1,52) | 0.872 |
|  | Thalamus R | 0.225 | 0.289 | 0.504 | 0.130 | 0.125 | 3.542 (3,53) | 0.156 | 0.001 | 0.027 (1,52) | 0.870 |
|  | Substantia nigra L | 0.008* | 0.013* | <0.001* | 0.514 | 0.487 | 18.718 (3,53) | <0.001* | <0.005 | 0.001 (1,52) | 0.981 |
|  | Substantia nigra R | 0.005* | 0.003* | 0.002* | 0.549 | 0.523 | 21.487 (3,53) | <0.001* | 0.001 | 0.132 (1,52) | 0.718 |
|  | STN L | 0.208 | 0.136 | 0.085 | 0.210 | 0.165 | 4.692 (3,53) | 0.006* | 0.029 | 2.013 (1,52) | 0.162 |
|  | STN R | 0.056 | 0.044 | 0.044 | 0.348 | 0.298 | 9.275 (3,53) | <0.001* | 0.003 | 0.277 (1,52) | 0.601 |
|  | Red nucleus L | 0.942 | 0.043 | 0.278 | 0.297 | 0.257 | 7.459 (3,53) | <0.001* | 0.002 | 0.168 (1,52) | 0.684 |
|  | Red nucleus R | 0.044 | 0.172 | 0.533 | 0.343 | 0.293 | 8.530 (3,53) | <0.001* | 0.343 | 1.391 (1,52) | 0.244 |
|  | Cerebellar dentate L | 0.105 | 0.944 | 0.364 | 0.110 | 0.060 | 2.184 (3,53) | 0.101 | 0.006 | 0.324 (1,52) | 0.572 |
|  | Cerebellar dentate R | 0.256 | 0.303 | 0.845 | 0.157 | 0.109 | 3.295 (3,53) | 0.127 | 0.022 | 1.379 (1,52) | 0.246 |
| R2* | Caudate L | 0.050* | 0.184 | 0.765 | 0.295 | 0.256 | 7.410 (3,53) | <0.001* | 0.003 | 0.210 (1,52) | 0.649 |
|  | Caudate R | 0.034* | 0.272 | 0.679 | 0.288 | 0.247 | 7.133 (3,53) | <0.001* | 0.006 | 0.477 (1,52) | 0.493 |
|  | Putamen L | 0.063 | 0.773 | 0.517 | 0.162 | 0.115 | 3.426 (3,53) | 0.024* | 0.003 | 0.216 (1,52) | 0.644 |
|  | Putamen R | 0.060 | 0.872 | 0.646 | 0.163 | 0.116 | 3.450 (3,53) | 0.023* | 0.005 | 0.295 (1,52) | 0.589 |
|  | Pallidum L | 0.049* | 0.459 | 0.570 | 0.230 | 0.186 | 5.264 (3,53) | 0.003* | <0.005 | 0.012 (1,52) | 0.915 |
|  | Pallidum R | 0.041* | 0.522 | 0.636 | 0.221 | 0.177 | 5.010 (3,53) | 0.004* | <0.005 | 0.018 (1,52) | 0.895 |
|  | Thalamus L | 0.071 | 0.993 | 0.509 | 0.126 | 0.076 | 2.536 (3,53) | 0.067 | <0.005 | 0.029 (1,52) | 0.866 |
|  | Thalamus R | 0.145 | 0.598 | 0.760 | 0.131 | 0.082 | 2.667 (3,53) | 0.057 | 0.001 | 0.060 (1,52) | 0.807 |
|  | Substantia nigra L | 0.018 | 0.013 | <0.005 | 0.082 | 0.030 | 1.586 (3,53) | 0.204 | 0.008 | 0.433 (1,52) | 0.514 |
|  | Substantia nigra R | 0.228 | 0.719 | 0.710 | 0.090 | 0.038 | 1.741 (3,53) | 0.170 | 0.004 | 0.235 (1,52) | 0.630 |
|  | STN L | 0.075 | 0.996 | 0.424 | 0.128 | 0.079 | 2.592 (3,53) | 0.062 | <0.005 | 0.004 (1,52) | 0.953 |
|  | STN R | 0.100 | 0.883 | 0.493 | 0.095 | 0.044 | 1.855 (3,53) | 0.149 | <0.005 | 0.007 (1,52) | 0.934 |
|  | Red nucleus L | 0.259 | 0.997 | 0.564 | 0.058 | 0.005 | 1.091 (3,53) | 0.361 | 0.005 | 0.272 (1,52) | 0.604 |
|  | Red nucleus R | 0.232 | 0.892 | 0.510 | 0.055 | 0.002 | 1.033 (3,53) | 0.386 | 0.009 | 0.480 (1,52) | 0.491 |
|  | Cerebellar dentate L | 0.100 | 0.128 | 0.508 | 0.251 | 0.209 | 5.920 (3,53) | 0.100 | 0.009 | 0.630 (1,52) | 0.431 |
|  | Cerebellar dentate R | 0.124 | 0.487 | 0.805 | 0.158 | 0.110 | 3.319 (3,53) | 0.027 | 0.001 | 0.033 (1,52) | 0.856 |

Table 3: Summary of the linear models explaining the mean susceptibility (χ) and R2*. The models were estimated separately for each deep brain structure and each map. The model predictors were the subject’s age, disease presence, disease duration and interaction between the disease effect and age. We report the p-value of each predictor, the determination coefficient (R^2^), adjusted R^2^ (adj R^2^), F-value and p-value for each model without the interaction term. Additionally, we report the R^2^ change, F-value change and p-value of the model with the interaction between the disease effect and age. STN = sub-thalamic nucleus, L=left, R= right, * indicates a significant (p < 0.05) model - predictor.


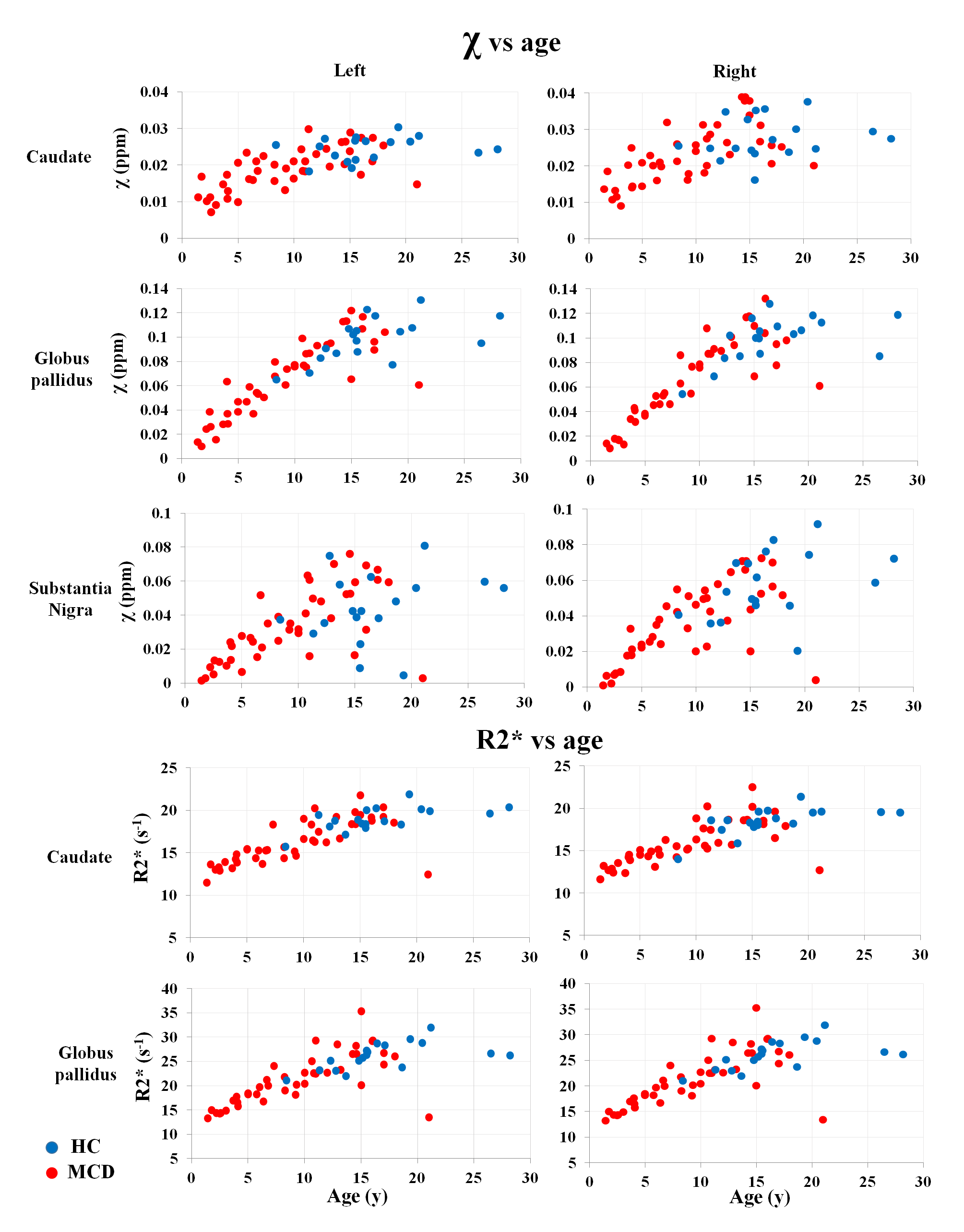


Figure 7: Linear correlation between age and susceptibility (χ), R2* in deep brain nuclei. The graphs show the mean χ and R2* values in left and right caudate, globus pallidus and substantia nigra, plotted against age for the malformation of cortical development (MCD) patients (red) and healthy controls (HC) (blue). The plots are shown only for the deep brain nuclei for which we found age as significant predictor in the linear models.
